# Simple gene knockout by single gene-directed multiplex CRISPR-Cpf1

**DOI:** 10.1016/j.gendis.2025.101646

**Published:** 2025-04-21

**Authors:** Yeon-Ju Jeong, Gyeong-Nam Kim, Jeongin Cho, Young Hoon Sung

**Affiliations:** a Department of Medical Science and Asan Medical Institute of Convergence Science and Technology, Asan Medical Center, University of Ulsan College of Medicine, Seoul 05505, Republic of Korea; b Department of Cell and Genetic Engineering, Asan Medical Center, University of Ulsan College of Medicine, Seoul 05505, Republic of Korea; c Convergence Medicine Research Center, Department of Convergence Medicine, Biomedical Research Center, Asan Institute for Life Sciences, Asan Medical Center, Seoul 05505, Republic of Korea

The CRISPR-Cas system is a powerful genetic engineering tool and can be conveniently used for the generation of diverse gene-knockout models. One CRISPR-Cas system, CRISPR-Cpf1 (also known as Cas12a), recognizes the AT-rich protospacer-adjacent motif (PAM) present at the 5′ end of the target sequence and requires CRISPR RNA (crRNA), but not transactivating crRNA (tracrRNA) for its activity.^1^ Unlike Cas9, Cpf1 can produce multiple mature crRNAs by processing a concatemeric crRNA precursor and thus is useful for the multiplex gene targeting.^2^

Like Cas9, Cpf1-induced double-stranded breaks (DSBs) are primarily repaired in cells by non-homologous end-joining (NHEJ), which results in frame shifts by insertions and deletions (indels) and subsequent premature termination codons (PTC).^1^ However, one-third of indel mutations induced by individual crRNAs are in-frame, and thus these crRNAs alone cannot completely block protein expression. Therefore, after expressing Cpf1 and crRNA in cells, a considerable number of monoclonal cell lines must be prepared so that appropriate clones harboring PTCs in all alleles can be selected. However, this process not only entails considerable cost and labor, but also has multiple caveats. For example, as cancer cell lines normally express high level of clonal variation, separate clones in which the same gene is knocked out frequently have significant phenotypic differences.^3^ These phenotypic differences make it difficult to interpret the physiological significance of the results obtained using gene-knockout cancer cell clones. Moreover, the lifespan of primary cells is finite, limiting efficient production and use of gene-knockout clones.

In this study, we hypothesized that systematic use of multiplex CRISPR-Cpf1 for the targeting a single gene would minimize in-frame mutations, allowing the generation of a polyclonal isogenic cell population containing a minimal number of cells with in-frame mutations. Even if indel mutations are induced in all cells by a single crRNA, >50% of the cell population would still contain 1–2 in-frame mutant alleles. If the second crRNA produces additional frameshift mutations at the downstream target site without disrupting the pre-existing PTCs generated by the upstream crRNA (Fig. 1A), the cell population harboring in-frame mutant alleles will be considerably reduced. If this process is conducted repetitively using four mature crRNAs, it would be reasonable to anticipate that <3% of the resulting cell population would harbor in-frame mutations. As Cpf1 processes its own precursor crRNA,^2^ it can be simplified by expressing a precursor crRNA encoding four crRNAs (4CR) from a single construct (Fig. 1A).

**Figure 1:**
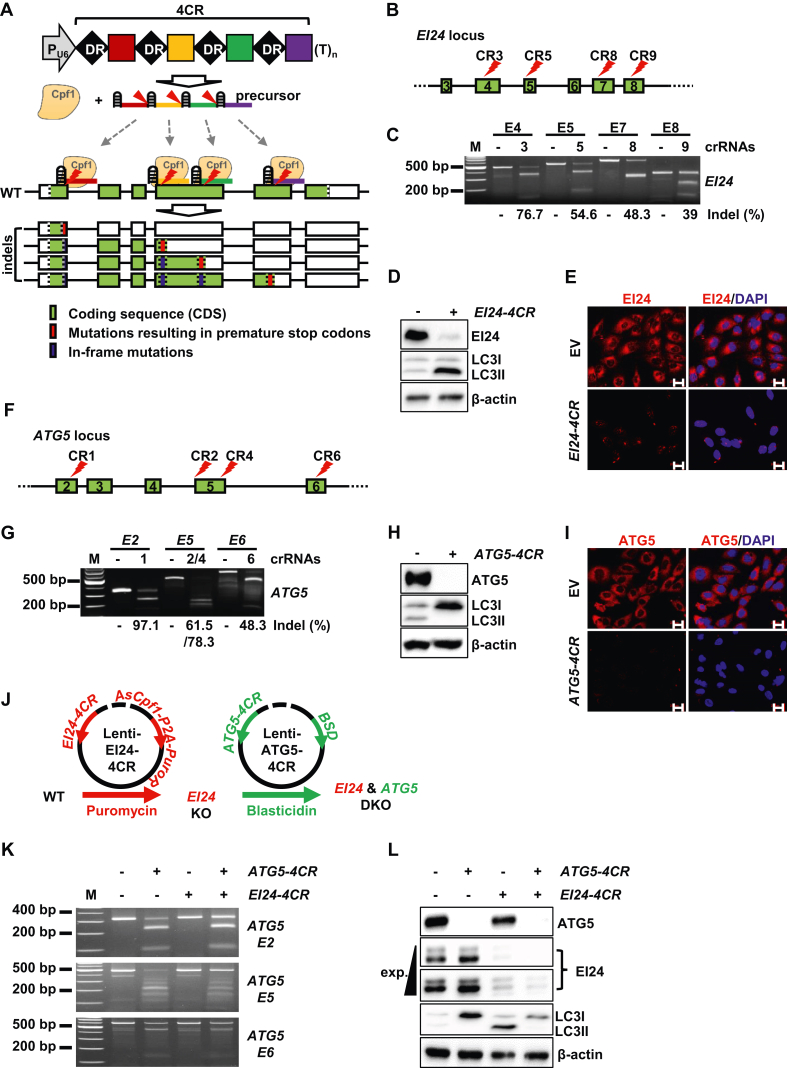
Generation of single gene and double gene-knockout cells using single gene-directed multiplex CRISPR-Cpf1. **(A)** Principle of single gene-directed multiplex CRISPR-Cpf1 used to serially enrich the gene-knockout cell population via the consecutive actions of four crRNAs. Downstream crRNAs cannot disrupt PTCs generated by upstream crRNAs. A precursor crRNA (4CR) encoding four mature crRNAs, each targeting a separate site in a single gene. **(B)** Genomic profile of the human *EI24* gene depicting approximate locations of crRNAs used to construct the *EI24* gene-specific 4CR construct (*EI24-4CR*). **(C**–**E)** Evaluation of *EI24-4CR*-mediated gene knockout in the SNU475 hepatocellular carcinoma cell line by T7E1 assays (C), Western blot analyses (D)**,** and immunofluorescence microscopy (E). **(F)** Genomic profile of the human *ATG5* gene depicting approximate locations of crRNAs used to construct the *ATG5* gene-specific 4CR construct (*ATG5-4CR*). **(G**–**I)** Evaluation of *ATG5-4CR*-mediated gene knockout in the SNU475 hepatocellular carcinoma cell line by T7E1 assays (G), Western blot analyses (H)**,** and immunofluorescence microscopy (I). **(J)** A diagram illustrating sequential application of *ATG5*-and *EI24*-specific 4CRs. *EI24*-targeted SNU475 cells were infected with lentivirus expressing *ATG5-4CR* to generate SNU475 cells lacking both *ATG5* and *EI24*. P2A, a 2A peptide derived from porcine teschovirus-1 2A; PuroR, puromycin N-acetyltransferase; BSD, blasticidin-S deaminase. (**K, L)** T7E1 assays of the *ATG5* gene (K) and Western blot analyses of ATG5 and EI24 proteins (L) to confirm *ATG5-4CR*-mediated gene knockout in the *EI24*-targeted SNU475 cells. The denoted indel frequencies (Indel [%]) were measured by amplicon deep-seq. LC3 proteins (LC3I and LC3II) were used as autophagy markers and β-actin was used as a loading control. Nuclei were counterstained with DAPI (4′,6-diamidino-2-phenylindole). Scale bar: 20 μm. M, a DNA molecular size marker (100-bp DNA ladder).

To investigate whether this system is efficient for gene knockout, the Cpf1 orthologue from Acidaminococcus sp. BV3L6 (AsCpf1) was employed.^1^ EI24 autophagy associated transmembrane protein (*EI24*) and autophagy related 5 (*ATG5*) genes are essential for the normal progression of autophagy.^4^ We designed a series of crRNAs specific for the human *EI24* gene and measured the indel activities of the individual crRNAs in SNU475 hepatocellular carcinoma cells (Fig. S1A). Each crRNA used in this study is listed in Supplementary Table S1. Highly active crRNAs, including CR3, CR5, CR8, and CR9, were selected for the generation of the *EI24*-specific 4CR construct (*EI24-4CR*; Fig. 1B; Fig. S1B). The oligomers and design strategy used for the 4CR construct are summarized in Supplementary Table S2 and Supplementary Figure S3. T7 endonuclease I (T7E1) assays and targeted amplicon sequencing analyses indicated that the *EI24-4CR* construct actively induced indel mutations in SNU475 cells (Fig. 1C). PCR primers used for T7E1 assay and sequencing are listed in Supplementary Table S3. The EI24 protein was barely detectable in SNU475 cells treated with *EI24-4CR*, as shown by Western blot (WB) and immunofluorescence (IF) analysis (Fig. 1D, E). Consistently, the LC3-phosphatidylethanolamine conjugate (LC3II), a well-known autophagy marker,^4^ accumulated in *EI24-4CR*-treated SNU475 cells, suggesting the inhibition of the autophagy flux (Fig. 1D). We also knocked out the *ATG5* gene using 4CR-mediated gene targeting. Detailed information is available in Supplementary Figure S3 and Table S1, S2, S3. Among the designed *ATG5*-specific crRNAs, CR1, CR2, CR4, and CR6 were highly active in SNU475 cells and were used to generate the *ATG5*-specific 4CR construct (*ATG5-4CR*; Fig. 1F; Fig. S1C, D). Notably, the target site of CR6 is located upstream of a possible PTC resulting from the frameshift (3n-1) induced by CR4-mediated indels. Nevertheless, *ATG5-4CR* efficiently induced indel mutations at their *ATG5* gene target sites (Fig. 1G), and the ATG5 protein was not detected in SNU475 cells (Fig. 1H, I). Furthermore, consistent with previous reports,^4^ 4CR-mediated *ATG5* gene knockout blocked the production of LC3II protein (Fig. 1H). These data demonstrate that the 4CR-mediated gene-knockout strategy eliminates the need to produce monoclonal cells for gene-knockout studies.

Unlike cancer cell lines, continuous in-vitro subculture of mortal primary cells leads to replicative senescence, severely limiting the establishment of gene-knockout clones. BJ normal human diploid fibroblasts have a finite lifespan.^5^ We observed that our 4CR expression vectors were useful for the establishment of *ATG5*-and *EI24*-deficient BJ cell cultures (Fig. S2A, B). As expected, while LC3II proteins were not detected in *ATG5* gene-deficient BJ cells, *EI24* gene knockout increased the level of LC3II proteins (Fig. S2C). These data indicate that the 4CR-mediated gene knockout can be performed in primary cells with a finite lifespan.

The successful gene knockouts using this novel strategy prompted us to generate cell lines deficient for both *ATG5* and *EI24* genes. When *EI24*-deficient SNU475 cells were infected again with a blasticidin (BSD)-resistant lentivirus expressing *ATG5*-specific 4CR (Fig. 1J, K), ATG5 protein expression was also abrogated (Fig. 1L). In support, LC3II protein did not accumulate in these *EI24*-deficient cells (Fig. 1L), since the production of LC3II protein is dependent on the upstream autophagy regulator, *ATG5* gene.^4^ Furthermore, concomitant treatment with *ATG5-4CR* and *EI24-4CR* efficiently abrogated the expression of both ATG5 and EI24 proteins in BJ normal human diploid fibroblasts (Fig. S2D-G). These results suggest that this method is also suitable for establishing double gene-knockout cells, which are useful for analyzing the genetic interactions between two genes.

In the present study, we demonstrate that our single gene-directed multiplex CRISPR-Cpf1 strategy is simple and robust not only for gene knockout in immortal cancer cell lines but also in mortal primary cells. Since this method does not require the establishment of monoclonal cells, various complications caused by clonal variations can be avoided: *i.e.*, the inherent characteristics of the parental cells are preserved in their gene-targeted isogenic cells generated using our single gene-directed multiplex CRISPR-Cpf1. We expect that this strategy and the validated 4CR expression constructs will help accelerate the pace of biomedical research and will be amenable to further development.

## CRediT authorship contribution statement

**Yeon-Ju Jeong:** Writing – review & editing, Writing – original draft, Validation, Methodology, Investigation. **Gyeong-Nam Kim:** Visualization, Validation, Methodology, Investigation, Formal analysis. **Jeongin Cho:** Visualization, Validation, Methodology, Investigation, Formal analysis. **Young Hoon Sung:** Writing – review & editing, Writing – original draft, Supervision, Investigation, Funding acquisition, Conceptualization.

## Funding

This work was supported by 10.13039/501100003725National Research Foundation of Korea (NRF) grants funded by the Ministry of Science, ICT, and Future Planning [2017M3A9C4065958 and 2018R1A2B6002192], by 10.13039/501100004080Korea Drug Development Fund (KDDF) funded by Ministry of Science and ICT, Ministry of Trade, Industry, and Energy, and Ministry of Health and Welfare [RS-2023-00283544], and by a grant from the Asan Institute for Life Sciences, Asan Medical Center, Seoul, Republic of Korea [2024IP0045].

## Conflict of interests

Y.H.S., Y.-J.J. and G.-N.K. have registered a patent application (10-2526994; Composition for manufacturing gene knock-out isogenic cell line comprising crRNA array and Cpf1 and use thereof) to the Korean Intellectual Property Office.
