# Supplementary material for: Simple gene knockout by single gene-directed multiplex CRISPR-Cpf1
Source: Genes Dis. 2025 Apr 21;13(2):101646. doi: 10.1016/j.gendis.2025.101646 (PMC12637181; doi:10.1016/j.gendis.2025.101646)
Supplement: Multimedia component 1 [file mmc1.docx]

**Simple gene knockout by single gene-directed multiplex CRISPR-Cpf1**

Yeon-Ju Jeong^1,2,3^, Gyeong-Nam Kim^1,2,3^, Jeongin Cho^1,2,3^, Young Hoon Sung^2,3*^

^1^Department of Medical Science and Asan Medical Institute of Convergence Science and Technology, Asan Medical Center, University of Ulsan College of Medicine, Seoul 05505, Republic of Korea.

^2^Department of Cell and Genetic Engineering, Asan Medical Center, University of Ulsan College of Medicine, Seoul 05505, Republic of Korea.

^3^Convergence Medicine Research Center, Department of Convergence Medicine, Biomedical Research Center, Asan Institute for Life Sciences, Asan Medical Center, Seoul 05505, Republic of Korea.

^*^Corresponding author: [yhsung@amc.seoul.kr](mailto:yhsung@amc.seoul.kr)

**Methods**

**Cell culture**

HEK 293TA cells (#LT008, GeneCopoeia), skin fibroblast cell BJ (#CRL-2522, ATCC), and hepatocellular carcinoma cell line SNU475 (#00475, Korean Cell Line Bank) were used in the experiments. All cell lines were maintained in complete medium supplemented with 10% fetal bovine serum and 1% penicillin-streptomycin at 37°C with 5% CO_2_ incubation.

**Vector construction and infectious lentiviral particle production**

Single crRNAs and multiplex crRNA arrays were cloned into the BsmBI sites of the pY108 lenti-AsCpf1 expression vector (#84739, Addgene). crRNAs specific for human *ATG5* and *EI24* genes were selected using Benchling (<https://www.benchling.com>). All oligomers used for experiments were purchased from Macrogen, Inc. (Seoul, Republic of Korea), and sequences are listed in Supplementary Tables 1 and 3.

To clone each multiplex crRNA array, a previously reported simple gene synthesis protocol was employed [1]. Briefly, three long oligomers encoding a multiplex crRNA array were ligated after annealing using two short linker oligomers and the ligated oligomers were then used as a template for the polymerase chain reaction (PCR; Figure S3). PCR products were subcloned into the BsmBI sites of the pY108 lenti-AsCpf1 expression vector and the LentiGuide-BSD vector, in which the U6 promoter drives transcription of the multiplex crRNA array. To construct the LentiGuide-BSD vector, the DNA fragment encoding the *blasticidin S deaminase* (BSD) gene was PCR-amplified from LentiGuide-blast (#104993, Addgene) using the following primers: 5′-ACGACCGGTATGGCCAAGCCTTTGTCTCA-3′ and 5′-GAAATTTGTGATGCTATTGC-3′ and was subcloned into the pY108 vector using the unique AgeI site and the SacII site in the SV40 polyadenylation signal. All constructs were verified by Sanger sequencing at Macrogen, Inc. (Seoul, Republic of Korea).

These vectors were then used to produce infectious lentiviral particles as described previously [2]. Blasticidin S hydrochloride and puromycin dihydrochloride purchased from Sigma-Aldrich Korea were used to select lentivirus-infected cells.

**Detection of indel mutations**

The genomic regions encompassing target sites were PCR-amplified from genomic DNA samples prepared from lentivirus-infected cells using the primers listed in Supplementary Table 3. To detect indel mutations, T7E1 assays were then conducted as described previously [3]. To estimate the molecular sizes of the DNA fragments, we used a 100-bp DNA ladder (#D-1030, BiONEER). To precisely measure the extent of indel mutations, targeted deep sequencing of PCR amplicons was performed using the Ilumina Miseq system at ToolGen (Seoul, Korea).

**Western blot analysis and immunofluorescence cytochemistry**

Protein samples were prepared by directly lysing cells in SDS sample buffer (#EBA-1052, Elpisbiotech) after washing the cells with ice-cold phosphate-buffered saline. To prepare samples for immunocytochemistry, cells were cultured on coverslips (#0111520, Marienfeld Superior).

Western blotting and immunocytochemistry were conducted using primary antibodies against ATG5 (#12994, Cells Signaling), EI24 (#HPA047165, Atlas Antibodies), LC3B (#3868, cell signaling), and β-actin (#SC-47778, Santa Cruz). Anti-rabbit IgG-HRP-linked antibody (#7074S, Cell Signaling) and anti-mouse IgG-HRP-linked antibody (#7076S, Cell Signaling) were used as secondary antibodies for Western blot analyses.

To detect fluorescence signals, Alexa fluor^TM^ 594 goat anti-rabbit IgG (H+L) (#A11012, Thermofisher) was used as the secondary antibody, and slides were mounted using DAPI mounting medium (#H-1200, Vector laboratories). Fluorescence microscopy was conducted using a Zeiss LSM 880 confocal microscope using a 40× water immersion objective.

**References**

1. Adams SE, Johnson ID, Braddock M, Kingsman AJ, Kingsman SM, Edwards RM. Synthesis of a gene for the HIV transactivator protein TAT by a novel single stranded approach involving in vivo gap repair. *Nucleic Acids Res*. 1988; 16: 4287-98.
2. Sung YH, Jin Y, Kang Y, Devkota S, Lee J, Roh JI, Lee HW. Ei24, a novel E2F target gene, affects p53-independent cell death upon ultraviolet C irradiation. *J Biol Chem.* 2013; 288: 31261-7.
3. Oh SH, Lee HJ, Ahn MK, Jeon MY, Yoon JS, Jung YJ, et al. Multiplex gene targeting in the mouse embryo using a Cas9-Cpf1 hybrid guide RNA. *Biochem Biophys Res Commun.* 2021; 539: 48-5

**Supplementary Table 1. Oligomers used to construct vectors expressing single crRNAs.**

| **Gene** | **crRNA name** | **Forward** | **Reverse** |
| --- | --- | --- | --- |
| *ATG5* | ATG5-CR1 | CCACAATCAATGTACTTACA | TGTAAGTACATTGATTGTGG |
|  | ATG5-CR2 | CAGAAAAAGACCTTCTGCAC | GTGCAGAAGGTCTTTTTCTG |
|  | ATG5-CR3 | AAGCATCAGCTTCTTTCATA | TATGAAAGAAGCTGATGCTT |
|  | ATG5-CR4 | CAATCCCATCCAGAGTTGCT | AGCAACTCTGGATGGGATTG |
|  | ATG5-CR5 | TATCATTACAGACAGATTTG | CAAATCTGTCTGTAATGATA |
|  | ATG5-CR6 | AAATGTTATTTCCTACCTGA | TCAGGTAGGAAATAACATTT |
| *EI24* | EI24-CR1 | GTGGTGAAGAGATGGCTGAC | GTCAGCCATCTCTTCACCAC |
|  | EI24-CR2 | TATAGGGAATCAAAGACTCC | GGAGTCTTTGATTCCCTATA |
|  | EI24-CR3 | CTGGATTCGAGCATCTAGCT | AGCTAGATGCTCGAATCCAG |
|  | EI24-CR4 | CAGTGAGCCACGTATTGTTA | TAACAATACGTGGCTCACTG |
|  | EI24-CR5 | CAGTGTTGTGCTTGGAATGG | CCATTCCAAGCACAACACTG |
|  | EI24-CR6 | CTCTTTCTTAGGTGACCCAT | ATGGGTCACCTAAGAAAGAG |
|  | EI24-CR7 | TTAGGTGACCCATCACTACA | TGTAGTGATGGGTCACCTAA |
|  | EI24-CR8 | GTCGTGGCTGGAATTCTTCC | GGAAGAATTCCAGCCACGAC |
|  | EI24-CR9 | TCTGTTACAGGATATAGCTG | CAGCTATATCCTGTAACAGA |
|  | EI24-CR10 | AGGTATCAGGGAGGAAGCCT | AGGCTTCCTCCCTGATACCT |

**Supplementary Table 2. Oligomers used to construct vectors expressing a multiplexed crRNA (4CR).**

| **Gene** | **Primer name** | **Sequence** |
| --- | --- | --- |
| *ATG5* | ATG5 CR-array-R1 | TCTTTTTCTGATCTACAAGAGTAGAAATTTGTAAGTACATTGATTGTGGATCTAGAGACGTAT |
|  | ATG5 CR-array-R2 | GGATGGGATTGATCTACAAGAGTAGAAATTGTGCAGAAG |
|  | ATG5 CR-array-R3 | AGACGTCTCAAAAATCAGGTAGGAAATAACATTTATCTACAAGAGTAGAAATTAGCAACTCT |
|  | ATG5 CR-Bridge-F1 | AGATCAGAAAAAGACCTTCTGCAC |
|  | ATG5 CR-Bridge-F2 | AGATCAATCCCATCCAGAGTTGCT |
|  | ATG5 PCR-F | ATACGTCTCTAGATCCACAATCAATGTACTTACA |
|  | ATG5 PCR-R | AGACGTCTCAAAAATCAGGTAGGAAATAACATTT |
| *EI24* | EI24 CR-array-R1 | TGGCTCACTGATCTACAAGAGTAGAAATTGGAGTCTTTGATTCCCTATAATCTAGAGACGTAT |
|  | EI24 CR-array-R2 | GGGTCACCTAAATCTACAAGAGTAGAAATTTAACAATACG |
|  | EI24 CR-array-R3 | AGACGTCTCAAAAAAGGCTTCCTCCCTGATACCTATCTACAAGAGTAGAAATTTGTAGTGAT |
|  | EI24 CR-Bredge-F1 | AGATCAGTGAGCCACGTATTGTTA |
|  | EI24 CR-Bredge-F2 | AGATTTAGGTGACCCATCACTACA |
|  | EI24 PCR-F | ATACGTCTCTAGATTATAGGGAATCAAAGACTCC |
|  | EI24 PCR-R | AGACGTCTCAAAAAAGGCTTCCTCCCTGATACCT |

**Supplementary Table 3. PCR primer pairs for T7E1 assays.**

| **Gene** | **Target sgRNA** | **Primer name** | **Forward** | **Reverse** | **Size (bp)** |
| --- | --- | --- | --- | --- | --- |
| *ATG5* | CR1 | ATG5-E1-F2/ATG5-E1-R1 | TGTGCTTCGAGATGTGTGGT | GTCCAGAACGCATCATGACA | 319 |
|  | CR2,3,4 | ATG5-E4-F2/ATG5-E4-R2 | GGGTTATTTCAGTGCTAAGAGATAG | CAGAGGACACCAAAAGAGCAG | 474 |
|  | CR5,6 | ATG5-E6-F1/ATG5-E6-R2 | CTGTACCTTTGTAGCTCAGCA | AAAGACACAGTTTGGAAAACCCC | 590 |
| *EI24* | CR3 | EI24-E4-F1/EI24-E4-R2 | AAGATACTCAGTACGTGGGTGG | TCCGCTCTATACTCTGGGCT | 469 |
|  | CR5 | EI24-E5-F1/EI24-E5-R1 | TGACTGACATTAGAACATTGGGAGA | CCTTCCCGAGTCCCCATAGTT | 534 |
|  | CR7 | EI24-E7-F1/EI24-E7-R1 | CCTGCAGATAGCGTACTGGT | ACCAGACACCTGCCAATGAG | 608 |
|  | CR9 | EI24-E8-F2/EI24-E8-R1 | GGCGGACTAGTGGCCTTA | AAAATCATTACCTCCCACCAGCAT | 333 |


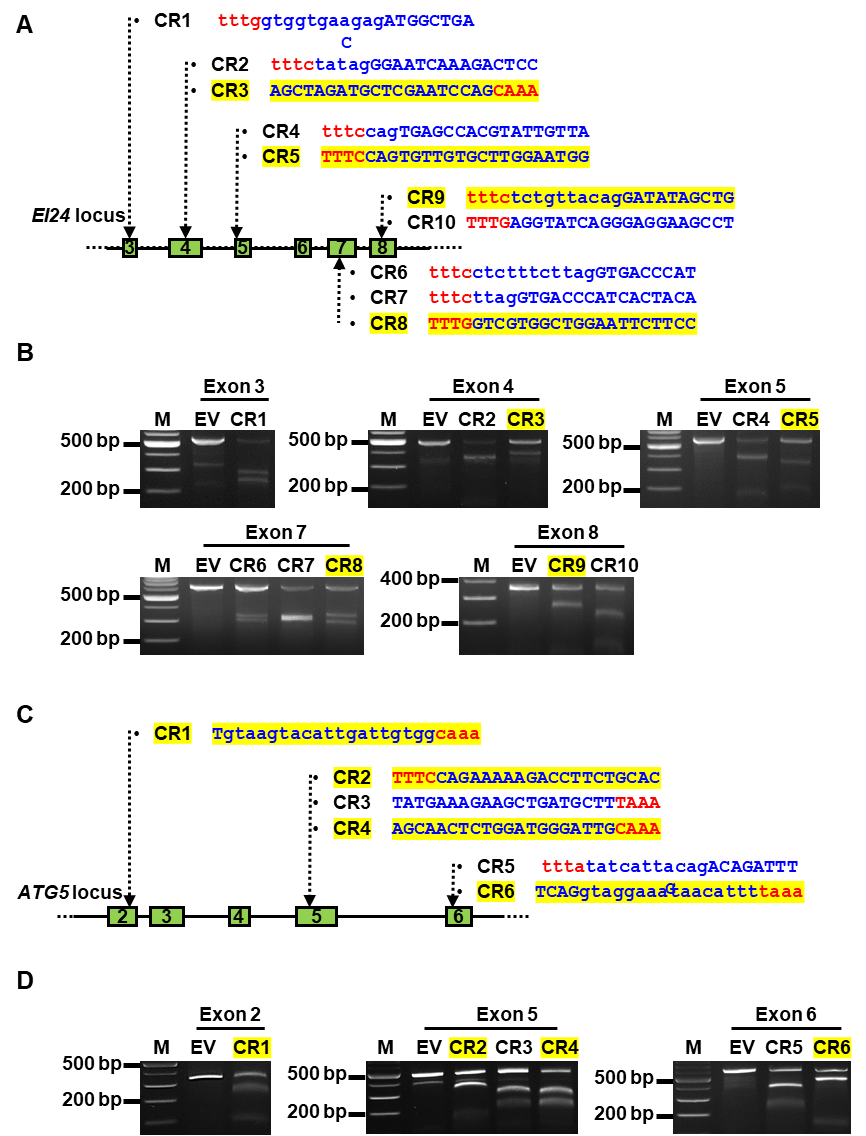


**Figure S1. Selection of highly mutagenic EI24 and ATG5-specific crRNAs in the SNU475 human hepatocarcinoma cell line.** **(A)** Human *EI24* locus showing crRNA target sequences. Uppercase sequences represent exons, and lowercase sequences represent introns. Spacers are denoted in blue, and PAMs are denoted in red. **(B)** T7E1 assays to measure indel mutations in the *EI24* gene induced by the indicated crRNAs. crRNAs highlighted in yellow were used to construct *EI24-4CR*. **(C)** Schematic genomic profile of the human *ATG5* locus with the crRNA target sequences highlighted. Uppercase sequences represent exons, and lowercase sequences represent introns. Spacers are denoted in blue and protospacer-adjacent motifs (PAMs) are denoted in red. **(D)** T7E1 assays to measure levels of indel mutations in the *ATG5* gene induced by the indicated crRNAs. The crRNAs highlighted in yellow were used to construct *ATG5-4CR*. M, a DNA molecular size marker (100-bp DNA ladder).


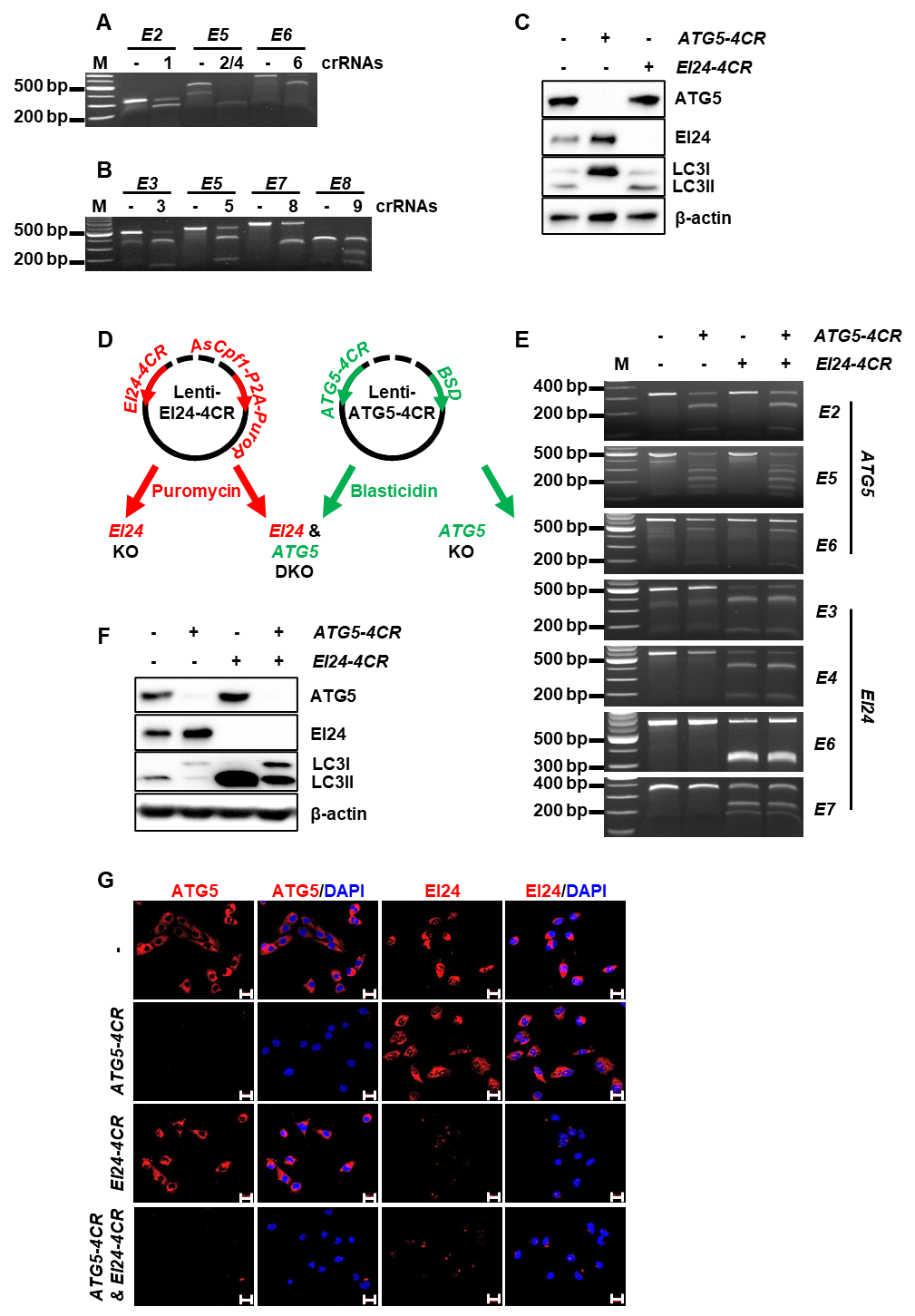


**Figure S2. Single and double gene knockout in BJ normal human cells using a single gene-directed multiplex CRISPR-Cpf1. (A, B)** T7E1 assays evaluating *ATG5-4CR*-mediated **(A)** or *EI24-4CR*-mediated **(B)** indel mutation levels in BJ cells. **(C)** Western blot analyses of ATG5 and EI24 protein levels in 4CR-treated cells. LC3 proteins (LC3I and LC3II) were used as autophagy markers and β-actin was used as a loading control. (**D**) Schematic describing the concomitant use of ATG5- and EI24-specific 4CRs for the generation of a cell population deficient in both ATG5- and EI24. PuroR, puromycin N-acetyltransferase; BSD, blasticidin S deaminase. (**E, F**) Western blot analyses (**E**) and T7E1 assays (**F**) of BJ normal human skin cells infected with lentiviruses expressing EI24-4CR or ATG5-4CR. (**G**) *ATG5-4CR*- and/or *EI24-4CR*-treated BJ normal human diploid fibroblasts of were stained with antibodies specific for ATG5 and EI24 proteins. Nuclei were counterstained using DAPI. Scale bar: 20 μm. M, a DNA molecular size marker (100-bp DNA ladder).


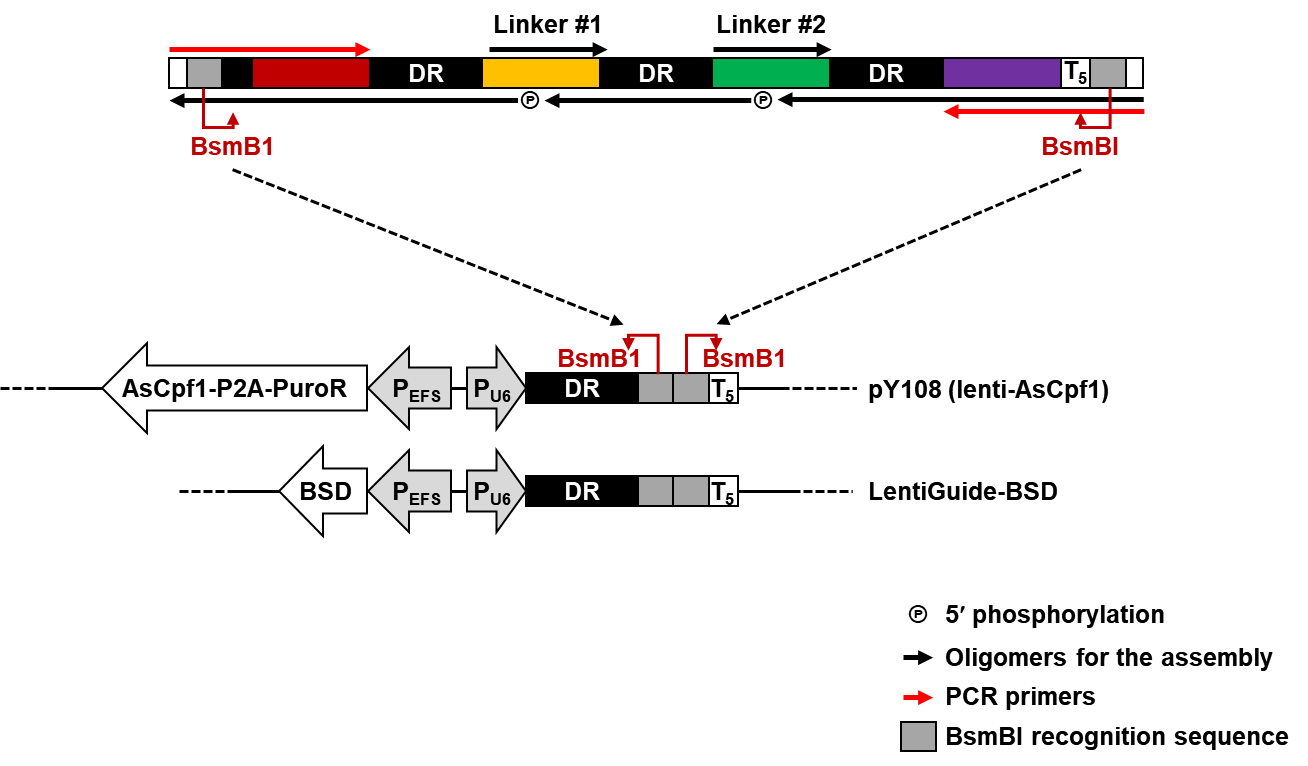


**Figure S3. Construction of the multiplex crRNA expression vector.** To generate DNA fragments harboring a multiplex crRNA array, three long oligomers were ligated using two short linker oligomers, and the ligated oligomer fragment was used as a PCR template. Each of the synthesized DNA fragments was digested with BsmBI restriction enzyme and subsequently subcloned into BsmBI sites of the pY108 lenti-AsCpf1 expression vector and LentiGuide-BSD vector in which the U6 promoter drives transcription of 4CR.
